# Clinical significance of dynamical network indices of surface electromyography for reticular neuromuscular control assessment

**DOI:** 10.1186/s12984-023-01297-3

**Published:** 2023-12-20

**Authors:** Jinping Li, Xianglian Kang, Ke Li, Ying Xu, Zhengfei Wang, Xinzhi Zhang, Qingjia Guo, Runing Ji, Ying Hou

**Affiliations:** 1 Department of Rehabilitation Medicine, Suzhou Municipal Hospital, Gusu School, Affiliated Suzhou Hospital of Nanjing Medical University, Nanjing Medical University, Suzhou, 215000 China; 2 Department of Medical Engineering, Suzhou Municipal Hospital, Gusu School, Affiliated Suzhou Hospital of Nanjing Medical University, Nanjing Medical University, Suzhou, 215000 China; 3 Laboratory of Rehabilitation Engineering, Intelligent Medical Engineering Research Center, School of Control Science and Engineering, Shandong University, Jinan, 250061 China; 4 Department of Rehabilitation Medicine, Changshu No.1 People’s Hospital, Changshu Affiliated Hospital of Soochow University, Changshu, 215500 China

**Keywords:** Gusu Constraint Standing Training, Multiplex recurrence network, Surface electromyography, Reticular neuromuscular control, Lower limbs, Motor model

## Abstract

**Background:**

There is currently no objective and accurate clinical assessment of reticular neuromuscular control in healthy subjects or patients with upper motor neuron injury. As a result, clinical dysfunctions of neuromuscular control could just be semi-quantified, efficacies and mechanisms of various therapies for neuromuscular control improving are difficult to verify.

**Methods:**

Fourteen healthy participants were required to maintain standing balance in the kinetostatics model of Gusu Constraint Standing Training (GCST). A backward and upward constraint force was applied to their trunk at 0°, 20° and 25°, respectively. The multiplex recurrence network (MRN) was applied to analyze the surface electromyography signals of 16 muscles of bilateral lower limbs during the tests. Different levels of MRN network indices were utilized to assess reticular neuromuscular control.

**Results:**

Compared with the 0° test, the MRN indices related to muscle coordination of bilateral lower limbs, of unilateral lower limb and of inter limbs showed significant increase when participants stood in 20° and 25° tests (*P* < 0.05). The indices related to muscle contribution of gluteal, anterior thigh and calf muscles significantly increased when participants stood in 20° and 25° tests (*P* < 0.05).

**Conclusions:**

This study applied the dynamical network indices of MRN to analyze the changes of neuromuscular control of lower limbs of healthy participants in the kinetostatics model of GCST. Results showed that the overall coordination of lower limb muscles would be significantly enhanced during performing GCST, partly by the enhancement of neuromuscular control of single lower limb, and partly by the enhancement of joint control across lower limbs. In particular, the muscles in buttocks, anterior thighs and calves played a more important role in the overall coordination, and their involvement was significantly increased. The MRN could provide details of control at the bilateral lower limbs, unilateral lower limb, inter limbs, and single muscle levels, and has the potential to be a new tool for assessing the reticular neuromuscular control.

*Trial registration* ChiCTR2100055090

**Supplementary Information:**

The online version contains supplementary material available at 10.1186/s12984-023-01297-3.

## Background

The central control of human movement is hierarchical and reticular [1]. Therefore, neuromuscular control dysfunctions of patients with upper motor neuron injury manifests not only as functional decreases in movement ability, but also as breakdown of neuromuscular network, or spatial and temporal coordination of muscles [1–3]. The functional recovery of these patients may not come from muscle activation and increased muscle strength, but rather from repeated postural and motor training that corrects motor pattern of multiple muscles, segments, and limbs. Neurodevelopment treatment, proprioceptive neuromuscular facilitation, and motor relearning training all belong to this treatment modality [4–6]. It is important to distinguish the networks of different neuromuscular control models and find their characteristics to enhance the therapeutic effects.

Even objective and accurate assessment methods, such as the surface electromyography (sEMG), can only be used to identify the sequence and degree of muscle activation, but not to assess the hierarchical pattern of neuromuscular control and multi-muscle coordination in clinic. As a result, clinical dysfunctions of neuromuscular control could just be semi-quantified, efficacies and mechanisms of various neuromuscular therapies are difficult to verify [7, 8]. Finding a method that can efficiently perform hierarchical neuromuscular control pattern recognition is of paramount importance.

This challenge is expected to be overcome by multiplex recurrence network (MRN), which is a novel nonlinear dynamical network algorithm [9–12]. In 2018, Deniz Eroglu et al. designed the MRN by combining recurrence characteristics with the multiplex network, and applied it to multivariate time series analysis [10]. The MRN algorithm transforms the original one-dimensional time series into state points of high-dimensional phase space, characterizes the recurrence features of subsystem through the spatiotemporal relationship of state points, and finally achieves the determination of synchronization adjustability of multi-subsystems by analyzing the similarity between recurrence features of subsystems. The MRN algorithm may provide a new way to distinguish the neuromuscular control models by identifying synchronization adjustability [10, 11]. Our team have applied the MRN to assess the inter-muscular coordination for both grip and pinch at different force levels, demonstrating that the type of grasp and the level of grasping force both had an impact on the coordination of hand muscles [12]. Although this method seemingly has some technical advantages, it is still not clear whether it could be applied in clinic to reflect the characteristics of reticular neuromuscular control or what clinical significances of its indices is.

In this study, we plan to use MRN to explore the changes in hierarchical neuromuscular control of healthy adults in an oriented muscle-activating kinetostatics model based on external-internal force coupling. The kinetostatics model in this study is derived from the Gusu Constraint Standing Training (GCST) designed by our team [13, 14]. We chose the A_2_ step of GCST (GCST-A_2_) as the test model. In this model, a backward and upward constraint force applied to the trunk of the participants can correspondingly activate the anterior muscles and inhibit the posterior muscles of lower limbs. Previous studies have demonstrated that this treatment could improve strength of lower limbs of patients with cerebral palsy, optimize the morphology of the gluteus maximus muscles, and fix knee hyperextension, anterior pelvic tilt, and crouch gait [13, 14].

## Methods

### Participants

Fourteen healthy participants aged between 20 and 40 years were recruited. Participants with (1) a history of musculoskeletal injuries on their lower limbs; (2) lower limb pain; (3) severe cervical or lumbar spine diseases; (4) dizziness or vestibular diseases; and (5) severe visual impairment, were excluded from this study. Ethics Committee of the Affiliated Suzhou Hospital of Nanjing Medical University approved this study according to the Declaration of Helsinki (K-2022-161-K01). All participants signed the informed consent. The characteristics of participants are detailed in Table 1.

**Table 1:** The characteristics of participants ($\stackrel{-}{x}$ ± SD)

Sex (male / female)	Age (year)	Height (cm)	Weight (kg)
6 / 8	25.29 ± 5.85	168.54 ± 7.42	63.21 ± 13.20

### Procedures

The 16-channel wireless sEMG system (Trigno™, Delsys, USA) was utilized to record the physiological electrical signals of muscles, and the sampling frequency was set at 2000 Hz. Before the test, the skin of relevant electrode attachment sites was shaved, polished with scrubbing cream, and cleaned with alcohol. The non-invasive wireless silver-contact bipolar bar electrodes of sEMG system, were attached to the muscle belly of bilateral gluteus maximus (GMA), gluteus medius (GME), vastus medialis (VM), vastus lateralis (VL), biceps femoris (BF), tibial anterior (TA), gastrocnemius (GM), and soleus (SL) by double-sided adhesive. After the sEMG electrodes were prepared, the participants wore the sling and stood in a specific position with the help of the testers. A scale under the participant’s feet was used to record the weight reduction during test.

**Fig. 1:**
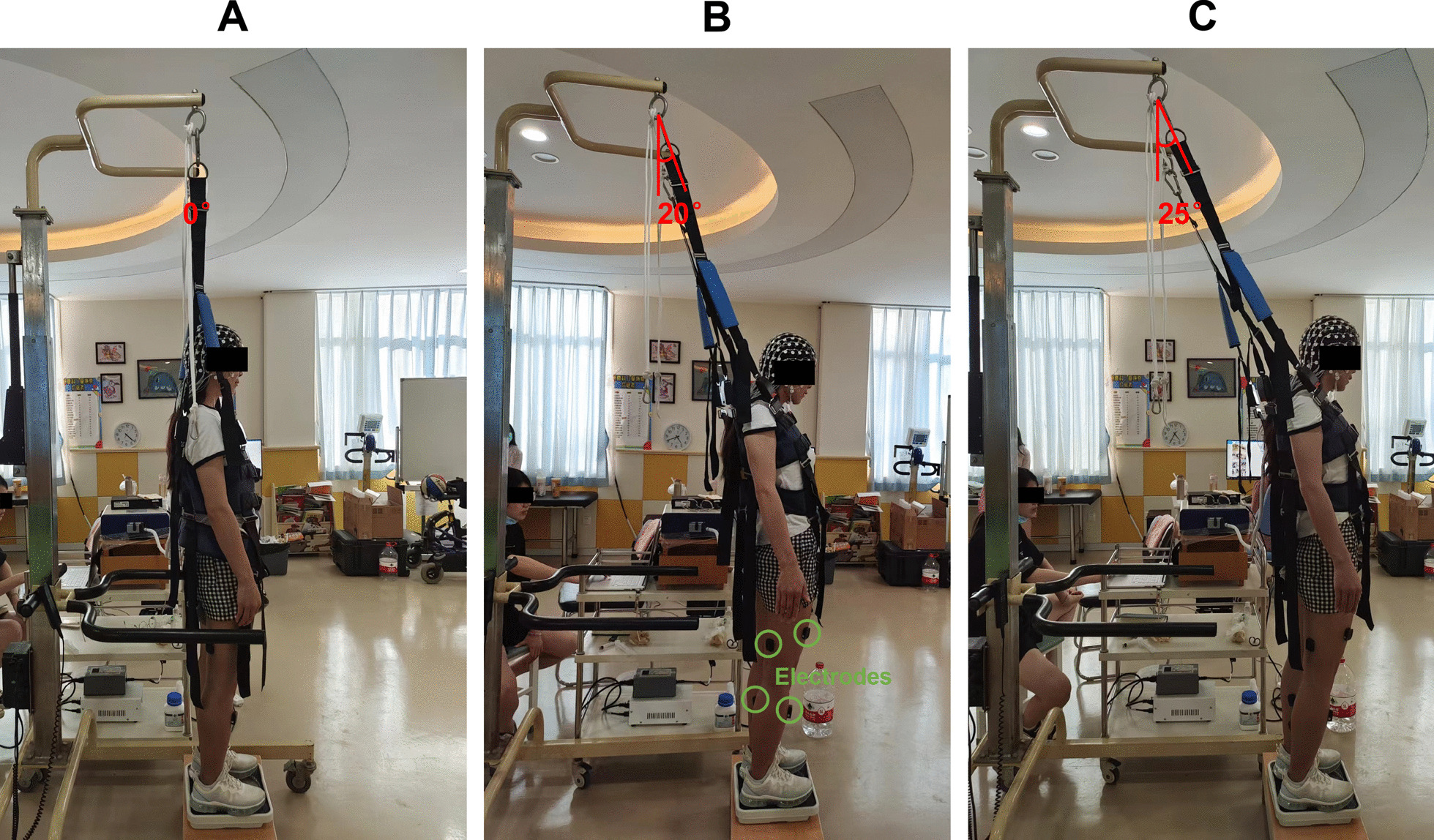
One participant performed standing tests under three conditions. A: 0° test; B: 20° test; C: 25° test

Three tests were set up, including standing with no constraint force (0° test), with constraint force at small angle (20° test), and with constraint force at large angle (25° test). Among them, the 20° and 25° tests met the setting requirements of GCST-A_2_. In 0° test, the belts sagged naturally and exerted no constraint force on the participants. In 20° and 25° tests, the testers adjusted the position and height of the body weight support device backward and upward to make the belts at angles of 20° and 25° from the vertical direction, respectively. Then, the testers gradually tightened the belts just before the participants could not maintain balance. In this state, the participants were standing with constraint forces backward and upward at 20° and 25°, respectively (Fig. 1).

During each test, the participants kept the upright standing position for 20 s with feet shoulder-width apart, hands hanging down naturally, and eyes looking straight ahead. Every test was repeated 3 times. The participants were allowed to have a 1 min rest between two tests.

### Data analysis

The MATLAB R2020a (The Mathworks, Natick, MA, USA) was used for data processing and analysis. There were 3 trials for each of the 0° test, 20° test and 25° test. The middle 15 s of the sEMG signals acquired in each trial was retained and band-pass filtered at 20 to 500 Hz using a fourth-order Butterworth filter. A sliding window with 1000 samples and an overlap of 250 samples was applied to the construction of MRN and extraction of indices. The average value of indices obtained from all sliding windows was taken as the result of one trial. The average of the indices extracted from the signals collected over 3 trials of one test was used as the final test result for that participant.

**Fig. 2:**
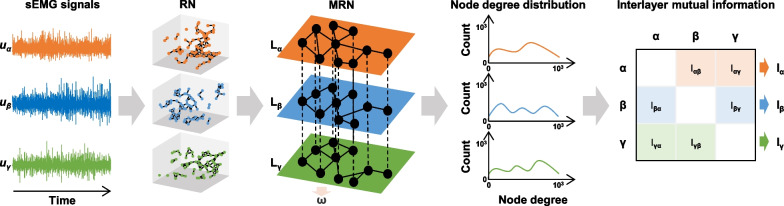
Schematic of the MRN construction and the indices extraction

The muscle network was constructed using a nonlinear dynamical network method MRN [10, 12]. Firstly, each channel sEMG time series was reconstructed as a trajectory in high-dimensional phase space [15]. For phase space reconstruction, the time delay was set at 5 samples using mutual information, and the embedding dimension was set at 4 using false nearest neighbors [16, 17]. Then, the recurrence network (RN) of single-channel sEMG time series could be obtained by comparing the distance between the state points of the reconstructed trajectory and the threshold value. If the distance between two state points was less than or equal to the threshold, there was a connection between two corresponding nodes of RN. Conversely, if the distance between two state points was greater than the threshold, there was no connection between two corresponding nodes of RN. The threshold was set at 80% of the maximum phase space radius according to our previous research [18]. The cross-recurrence plot toolbox 5.1 of MATLAB was used to construct the RN. Finally, placing the RN of single-channel sEMG time series into the associated layer and connecting layers with corresponding nodes, the MRN of multi-channel sEMG time series was created (Fig. 2).

The dynamical coordination of multiple muscles can be described quantitatively by extracting relevant indices of MRN. The interlayer mutual information *I*_*αβ*_ between *α* and *β* can be used to capture the presence of inter-layer similarity of node degree distribution (Fig. 2), whose calculation formula is as follows:

1 $${I_{\alpha \beta }}{\text{=}}\sum\limits_{{{k^{\left[ \alpha \right]}}}} {\sum\limits_{{{k^{\left[ \beta \right]}}}} {P\left( {{k^{\left[ \alpha \right]}},{k^{\left[ \beta \right]}}} \right)} } \log \frac{{P\left( {{k^{\left[ \alpha \right]}},{k^{\left[ \beta \right]}}} \right)}}{{P\left( {{k^{\left[ \alpha \right]}}} \right)P\left( {{k^{\left[ \beta \right]}}} \right)}}$$

where *P*(*k*^[*α*]^) and *P*(*k*^[*β*]^) are the degree distribution probabilities of layers *α* and *β*, respectively. The *P*(*k*^[*α*]^,*k*^[*β*]^) is the joint degree distribution probability of the existence of nodes with degree *k*^[*α*]^ in layer *α* and *k*^[*β*]^ in layer *β*.

The Fig. 2 exhibits the interlayer mutual information between all possible inter-layer pairs in MRN. The average interlayer mutual information (*I*) could be obtained by averaging the mutual information between selected muscle pairs. In this study, the *I* of bilateral lower limbs was the average of the mutual information between all muscle pairs. The *I* of left lower limb and of right lower limb were the averages of the mutual information between pairs of muscles belonging to the same side, respectively. The inter-limb *I* was the average of the mutual information between pairs of muscles belonging to different sides. The higher the *I* is, the more similar the dynamical structure between muscles’ activation is.

In order to quantify the dynamic-structural coupling of muscle *α* with other muscles, the mutual information between muscle *α* and other muscles was summed to obtain muscle-related *I* of *α*, as shown in the rightmost panel of Fig. 2. The larger the value of muscle-related *I*, the higher the involvement of this muscle in the overall muscle coordination.

Average edge overlap (*ω*) can be used to measure the average number of identical edges over m-layers of MRN, which is derived as:

2 $$\omega {\text{=}}\frac{{\sum\limits_{i} {\sum\limits_{{j>i}} {\sum\limits_{\gamma } {A_{{ij}}^{{\left[ k \right]}}} } } }}{{m\sum\limits_{i} {\sum\limits_{{j>i}} {\left( {1 - {\delta _{0,\sum\nolimits_{\gamma } {A_{{ij}}^{{\left[ k \right]}}} }}} \right)} } }}$$

where *A*_*ij*_ is the adjacency matrix of the single muscle RN, $i,j=1, \ldots ,N$, *N* is the length of sEMG signals, *m* represents the total number of layers of MRN, and *δ* is the Kronecker delta symbol. Via the averaged existence of overlapping edges across layers, the *ω* can characterize the synchronization of the microscopic dynamical structure of multivariate sEMG signals. The increased *ω* refers to enhanced coincidence in the timing of action potentials discharged by motor neurons across muscles. In this study, the *ω* of bilateral lower limbs, of left lower limb, and of right lower limb could be obtained.

### Statistical analyses

The statistical analyses were performed by SPSS 26.0 (IBM Corp, Armonk, NY, USA). A Shapiro–Wilk test was applied to verify the normal distribution of the data. Weight reduction during 20° and 25° constraint standing was analyzed using paired *t*-test. One-way repeated measures analysis of variance (ANOVA) was used to compare differences among three test conditions for the *I* and *ω* of bilateral lower limbs, and inter-limb *I*. Two-way repeated measures ANOVA was applied to examine the differences of test conditions and sides for *I* and *ω* of left and of right lower limb, and muscle-related *I*. Post hoc tests with Bonferroni were used for all pairwise comparisons. *P* < 0.05 was considered statistically different.

## Results

All participants successfully completed the experiment without discomfort or withdrawal. Net weight reduction was 6.25 ± 2.33 kg (10.04 ± 3.67% of body weight) and 3.46 ± 1.90 kg (5.67 ± 3.30% of body weight) in 20° and 25° test, respectively. The weight reduction during 20° constraint standing was significantly greater than that during 25° constraint standing (*P* < 0.05).

### Network indices of bilateral lower limbs

**Fig. 3:**
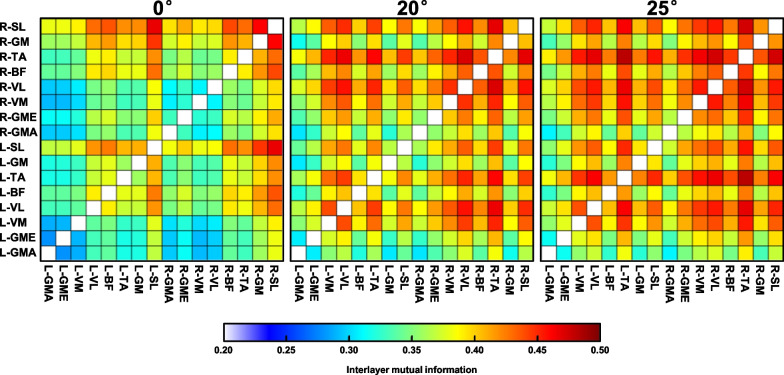
The interlayer mutual information matrix of MRN for a representative participant under different standing conditions. L: left limb; R: right limb

The interlayer mutual information matrix of MRN for a representative participant is illustrated in Fig. 3. Compared with the 0° test, the overall interlayer mutual information increased, and significantly larger values related to bilateral VM, VL and TA could be visually observed in the 20° and 25° tests.

**Fig. 4:**
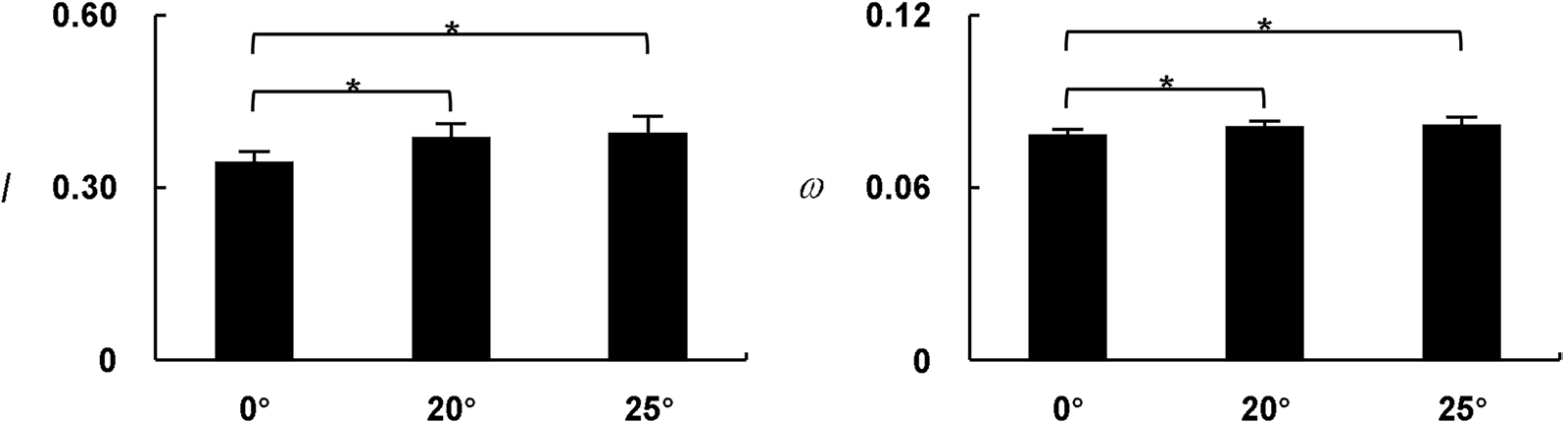
Network indices of bilateral lower limbs ($\stackrel{-}{x}$ ± SD). * Significant difference between two test conditions

Network indices of bilateral lower limbs were extracted and detailed in Fig. 4. The main effect of *test conditions* was found on *I* and *ω* (*I*: *F* = 21.136, *P* < 0.001; *ω*: *F* = 14.466, *P* < 0.001). Compared with the standing position without constraint force, the network indices of bilateral lower limbs in 20° and 25° constraint standing positions were significantly higher (*P* < 0.05). No significant differences were found between 20° and 25° tests (*P* > 0.05).

### Network indices of unilateral lower limb

**Fig. 5:**
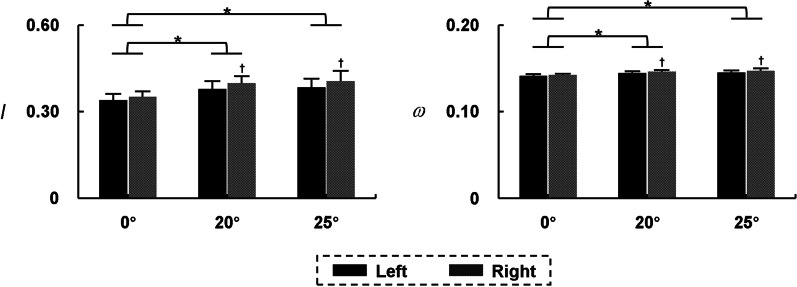
Network indices of unilateral lower limb ($\stackrel{-}{x}$ ± SD). * Significant difference between two test conditions; ^†^ Significant difference between two sides

The results of network indices of unilateral lower limb are shown in Fig. 5. An interaction effect between *test conditions* and *sides* was observed on *I* and *ω* (*I*: *F* = 8.861, *P* = 0.011; *ω*: *F* = 11.709, *P* = 0.005). When participants stood with constraint force at 20° and 25°, the network indices of both left and right limbs were significantly higher than those without constraint force (*P* < 0.05). There were no significant differences in the network indices of unilateral lower limb between the 20° and 25° constraint standing positions (*P* > 0.05). In addition, the network indices of right lower limb were significantly higher than those of left lower limb during constraint standing (*P* < 0.05).

**Inter-limb** ***I***.

**Fig. 6:**
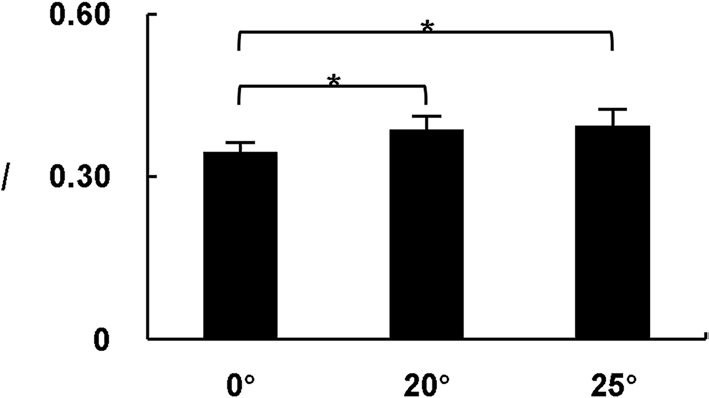
Statistical results of inter-limb *I* ($\stackrel{-}{x}$ ± SD). * Significant difference between two test conditions

Figure 6 depicts the results of the inter-limb *I*. There was a main effect of *test conditions* (*F* = 20.921, *P* < 0.001). Compared with the 0° test, the inter-limb *I* in 20° and 25° tests were significantly higher (*P* < 0.05). No significant differences of the inter-limb *I* were found between 20° and 25° tests (*P* > 0.05).

**Muscle-related** ***I***.

Table 2 shows the results of muscle-related *I*. The interaction effect between *test conditions* and *sides* was only observed on GMA-related *I* (*F* = 8.041, *P* = 0.014). Compared with the 0° test, the GMA-related *I* in 20° and 25°tests were significantly higher (*P* < 0.05). In constraint standing positions, the GMA-related *I* of left lower limb was significantly lower than that of right lower limb (*P* < 0.05). The main effect of *sides* was observed on muscles GME, VM, VL and TA (GME: *F* = 13.774, *P* < 0.001; VM: *F* = 81.945, *P* < 0.001; VL: *F* = 46.912, *P* < 0.001; TA: *F* = 64.175, *P* < 0.001). Compared with the static standing position, the GME-, VM-, VL-, and TA-related *I* were significantly greater in 20° and 25° constraint standing positions (*P* < 0.05). No significant difference of muscle-related *I* was found between the 20° and 25° constraint standing positions (*P* > 0.05).

**Table 2:** Statistical results of muscle-related *I* ($\stackrel{-}{x}$± SD)

Muscle	Left limb			Right limb		
	0°	20°	25°	0°	20°	25°
GMA	4.69 ± 0.21	5.09 ± 0.23^**a**^	5.13 ± 0.26^**a**^	4.77 ± 0.24	5.25 ± 0.37^**ab**^	5.30 ± 0.41^**ab**^
GME	4.82 ± 0.22	5.40 ± 0.51^**a**^	5.48 ± 0.56^**a**^	4.88 ± 0.28	5.59 ± 0.61^**a**^	5.75 ± 0.80^**a**^
VM	4.88 ± 0.27	6.08 ± 0.57^**a**^	6.13 ± 0.48^**a**^	4.91 ± 0.24	6.04 ± 0.45^**a**^	6.20 ± 0.48^**a**^
VL	4.99 ± 0.54	6.26 ± 0.43^**a**^	6.32 ± 0.47^**a**^	4.96 ± 0.45	6.32 ± 0.71^**a**^	6.35 ± 0.74^**a**^
BF	5.21 ± 0.71	5.49 ± 0.38	5.64 ± 0.42	5.22 ± 0.59	5.68 ± 0.55	5.77 ± 0.55
TA	5.03 ± 0.38	6.25 ± 0.33^**a**^	6.45 ± 0.45^**a**^	4.99 ± 0.30	6.44 ± 0.53^**a**^	6.65 ± 0.71^**a**^
GM	5.54 ± 0.74	5.46 ± 0.59	5.59 ± 0.73	5.94 ± 0.84	5.59 ± 0.69	5.76 ± 0.87
SL	6.07 ± 0.55	5.98 ± 0.61	6.09 ± 0.76	6.15 ± 0.45	6.23 ± 0.64	6.20 ± 0.76

^a^Significant difference compared with 0° test

^b^Significant difference compared with the left lower limb

**Fig. 7:**
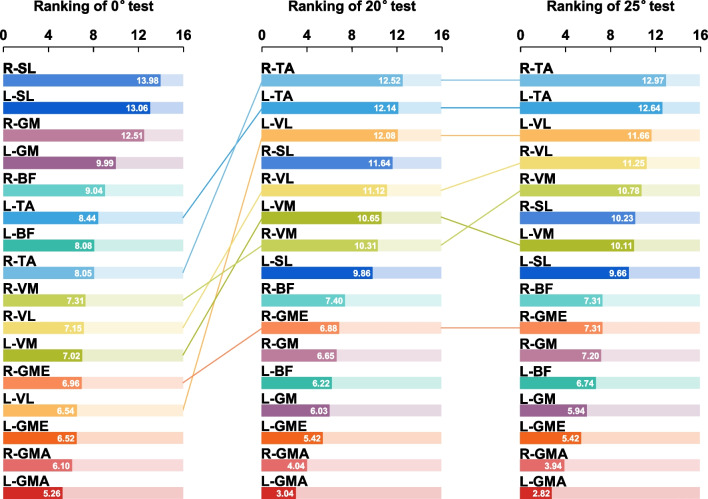
Ranking of muscle-related *I* of participants in three conditions

We also ranked the muscle-related *I* of networks by its values in descending order from 1 to 16. The ranking of muscle-related *I* is exhibited in Fig. 7. Compared with the 0° test, the ranking position of bilateral TA, VL, VM, and right GME were significantly advanced in 20° and 25° tests. There was little change in the ranking position of these muscles between 20° and 25° tests.

## Discussion

This study is the first to apply the MRN, a nonlinear dynamical network algorithm, to analyze the changes of neuromuscular control of lower limbs of healthy participants in the kinetostatics model of GCST-A_2_ from the perspective of reticular neuromuscular control.

### Overall muscle coordination of bilateral lower limbs

Compared with the 0° test, the overall muscle coordination of bilateral lower limbs was significantly enhanced when participants stood in GCST-A_2_ model at 20° and 25°. However, no significant difference was found between 20° and 25° tests (Fig. 4). These results suggested that the GCST-A_2_ model at both 20° and 25° could successfully intervene the overall neuromuscular control pattern of lower limbs. The weight reduction in GCST-A_2_ model at 20° was almost twice as much in GCST-A_2_ model at 25°, but there was no significant difference in muscle coordination of bilateral lower limbs. This confirms that the effect of GCST on muscle coordination does not originate from weight reduction, but rather from the constraint force. In order to resist external force exerted on the trunk and maintain standing balance, participants voluntarily enhanced the overall muscle coordination of the lower limbs [12, 19, 20]. From previous studies, it appears that enhanced muscle coordination may result from synchronous activation of the motor neuron [12, 19, 20]. This finding is also consistent with the overall improvement of muscle strength and motor skills in children with cerebral palsy in our team’s previous GCST efficacy study [13, 14]. The overall network indices did not show significantly differences under the GCST-A_2_ models at different angles and in the same direction of external force. It suggested that the overall network indices might be impacted less by the degree of angle of the external force than its direction to the body. The overall network indices *I* and *ω* of bilateral lower limbs are the results of dynamical coordination of all muscle activities, which could reflect degrees of synchronized regulation of overall neuromuscular control [21]. It is important to note that even if the values of network indices for two MRNs are close to each other, the dynamical coordination between the muscles within them is not the same. Therefore, it is essential to provide details of control at the unilateral lower limb, inter limbs, and single muscle level.

### Muscle coordination of unilateral lower limb

The results of network indices of unilateral lower limb showed that the GCST-A_2_ model would increase muscle coordination of each lower limb (Fig. 5). It may result from the symmetry requirement for balance of the participants. Previous studies have also demonstrated that children with cerebral palsy got symmetrical improvement in the GCST [13]. In addition, the level of synchronized regulation of neuromuscular control was significantly higher in the right lower limb than in the left lower limb (Fig. 5), which may result from the higher control skills and greater adaptability of the right lower limb [22–24]. During GCST-A_2_ tests, the right lower limb utilized its inherent motor control potential to a greater extent, maintaining overall postural stability through enhanced muscle coordination. In terms of the clinical significance, the network indices of the unilateral lower limb could not only reflect the state of neuromuscular control of the unilateral limb, but also analyze the level of symmetry of bilateral voluntary control.

### Muscle coordination between lower limbs

The results of inter-limb *I* showed that the muscle coordination between two lower limbs enhanced when the participants stood in GCST-A_2_ at 20° and 25°. However, no difference was found between two constraint standing conditions (Fig. 6). The results indicated that the participants could maintain overall stability by enhancing the dynamical coupling of two lower limbs when disturbed by external force. This change reflects the synergistic control of normal human bilateral movements realized through the brain corpus callosum and brainstem reticular formation [25, 26]. Therefore, the inter-limb *I* may be able to quantitatively assess the degree of association between bilateral limb motor control.

### Involvement of single muscle

The muscle-related *I* of muscles in buttocks, anterior thighs and calves during 20° and 25° tests were significantly increased, especially for GMA, GME, VM, VL and TA (Table 2; Fig. 7). These results suggested that the GCST-A_2_ model could activate muscles in buttocks, anterior thighs and calves, enabling them to have higher involvement and play a greater role in the new model of neuromuscular control. In the mechanical model GCST-A_2_, the participant’s body was subjected to a rearward and upward constraint. In order to resist the instability caused by this constraint force, gluteal muscles and anterior muscles of the lower limbs would activate synchronously to extend, erect and maintain the hip and knee joints, and work together to pull the body forward on the axis of the ankle joint. This is consistent with previous studies in which GCST can change the cross-sections morphology of gluteal muscle cell and improve muscle strength in the anterior muscles of the lower limbs [13].

In order to better interpret and compare the effectiveness of MRN in assessing neuromuscular control, we used non-negative matrix factorization (NMF) [27, 28], a state-of-the-art approach for muscle synergy description, to analyze the sEMG data collected in this study. The relevant results are presented in Additional file 1. The results of NMF showed an increased contribution of left VL and bilateral TA in the 20° and 25° tests compared to 0° test. This is consistent with the results using MRN. In addition to this, we found that the four muscle synergy modules obtained using NMF were very similar, suggesting that some information may been submerged using NMF. For example, the results of MRN showed a significant increase in the contribution of bilateral GMA, GME, and VM in the GCST-A_2_ model, whereas no such change was detected in the NMF results. The possible reason for this phenomenon is that the NMF performs muscle synergy module decomposition based on the changes of sEMG signal amplitude over a period. However, the subjects in this study received instructions to maintain standing stability, and the sEMG signal hardly had significant changes in amplitude. This may lead to difficulties in reflecting the intrinsic information of muscle synergy in NMF. On the contrary, the MRN could better detect highly contributing muscles in multi-muscle coordination in this study, and has a great advantage in evaluating data under dynamic steady conditions.

### Limitations

There are some limitations of this study: (1) The number of participants was relatively small, and the conditions of dominant side, sex, and age have not considered. In subsequent studies, more participants need to be recruited and grouped. (2) The magnitude of the constraint force from the belt should be measured and recorded quantitatively.

## Conclusions

This study applied the dynamical network indices of MRN to analyze the changes of neuromuscular control of lower limbs of healthy participants in the kinetostatics model of GCST-A_2_. Results showed that the GCST-A_2_ could successfully intervene in the neuromuscular control patterns of the lower limbs. The overall coordination of lower limb muscles would be significantly enhanced during performing GCST-A_2_, partly by the enhancement of neuromuscular control of single lower limb, and partly by the enhancement of joint control across lower limbs. In particular, the muscles in buttocks, anterior thighs and calves played a more important role in the overall coordination, and their involvement was significantly increased. The MRN could provide details of control at the bilateral lower limbs, unilateral lower limb, inter limbs, and single muscle levels, and has the potential to be a new tool for assessing the reticular neuromuscular control.

### Supplementary Information

**Additional file 1: Figure S1.** Averaged synergies and their corresponding temporal activation patterns of NMF for 0° test. **Figure S2.** Averaged synergies and their corresponding temporal activation patterns of NMF for 20° test. **Figure S3.** Averaged synergies and their corresponding temporal activation patterns of NMF for 25° test.

## Data Availability

The datasets used and/or analyzed during the current study are available from the corresponding author on reasonable request.

## Abbreviations

sEMG.: Surface electromyography
MRN.: Multiplex recurrence network
GCST.: Gusu Constraint Standing Training
GMA.: Gluteus maximus muscle
GME.: Gluteus medius muscle
VM.: Vastus medialis muscle
VL.: Vastus lateralis muscle
BF.: Biceps femoris muscle
TA.: Tibial anterior muscle
GM.: Gastrocnemius muscle
SL.: Soleus muscle
RN.: Recurrence network
I.: Average interlayer mutual information
ω.: Average edge overlap
NMF.: Non-negative matrix factorization
