# Supplementary material for: Clinical significance of dynamical network indices of surface electromyography for reticular neuromuscular control assessment
Source: J Neuroeng Rehabil. 2023 Dec 20;20:170. doi: 10.1186/s12984-023-01297-3 (PMC10734060; doi:10.1186/s12984-023-01297-3)
Supplement: Supplementary file 1 — Additional file 1: Figure S1. Averaged synergies and their corresponding temporal activation patterns of NMF for 0° test. Figure S2. Averaged synergies and their corresponding temporal activation patterns of NMF for 20° test. Figure S3. Averaged synergies and their corresponding temporal activation patterns of NMF for 25° test. [file 12984_2023_1297_MOESM1_ESM.docx]

**Additional file 1**

In order to better interpret and compare the effectiveness of multiplex recurrence network (MRN) in assessing neuromuscular control, we used non-negative matrix factorization (NMF) [1, 2], a state-of-the-art approach for muscle synergy description, to analyze the surface electromyography (sEMG) data collected in this study. The sEMG of 16 muscles, including bilateral gluteus maximus (GMA), gluteus medius (GME), vastus medialis (VM), vastus lateralis (VL), biceps femoris (BF), tibial anterior (TA), gastrocnemius (GM), and soleus (SL) were synchronously acquired in 0° test, 20° test, and 25° test.

The sEMG signals need to be preprocessed before applying NMF for analysis. Firstly, the sEMG signals were rectified to obtain the absolute value. Then, a fourth-order Butterworth filter with a cutoff frequency of 15 Hz was used for low-pass filtering to extract the envelope of sEMG signals. Finally, we normalized the sEMG signals using the maximum value method. The muscle synergies of 16 lower-limb muscles in 0°, 20° and 25° tests were exacted using NMF.

The number of muscle synergy modules was determined by calculating the variance accounted for (VAF, ranges from 0 to 1) between the original and the reconstructed profiles [3]. The number of muscle synergies was initially set to 1 and then increased sequentially. For each trial, we selected the modules with the minimum number of synergies. This requires that both two selection criteria are met simultaneously: all muscles achieved 95% VAF, and addition of an extra module would not increase the VAF by more than 0.5%. The final number of muscle synergies was determined to be 4. Fig. S1~S3 shows the average synergies and temporal activation patterns of NMF for three test conditions.

Fig. S1 Averaged synergies and their corresponding temporal activation patterns of NMF for 0° test.

 Fig. S2 Averaged synergies and their corresponding temporal activation patterns of NMF for 20° test.

Fig. S3 Averaged synergies and their corresponding temporal activation patterns of NMF for 25° test.

From all 4 muscle synergies, it can be observed that the highly contributing muscles in 0° test were L-GMA, L-GME, L-VM, L-GM, L-SL, R-VM, R-VL, R-GM and R-SL. The highly contributing muscles in the 20° and 25° tests were L-GMA, L-GME, L-VM, L-VL, L-TA, L-GM, L-SL, R-VM, R-VL, R-TA, R-GM and R-SL. Compared to the 0° test, the contribution of bilateral TA and left VL increased in the 20° and 25° tests. This is consistent with our observation using MRN that the bilateral GMA, GME, VM, VL, and TA had significantly higher involvement in the 20° and 25° tests. Notably, the four muscle synergies decomposed by NMF were essentially the same, and the proportion of each muscle in the different muscle synergies was similar, suggesting that some information may been submerged. For example, the MRN indices for bilateral GMA, GME, and VM increased in 20° and 25° tests, whereas the weight of these muscles did not change significantly in NMF.

While searching for previous studies, we found that NMF is mainly applied to analyze muscle synergies in dynamic motion, such as reaching and grasping with the upper limbs, walking, squatting, ascending or descending stairs, and applying external disturbance during standing still [1, 2, 4, 5]. In these motion, the sEMG signals would fluctuate significantly with time. The NMF can effectively decompose the muscle synergy modules based on the sEMG signal fluctuations. However, the subjects in this study received instructions to maintain standing stability, and their sEMG signals were in a relatively stable state with little variation in both temporal and spatial dimensions. This is consistent with the absence of significant fluctuations in the temporal activation pattern of NMF. This may lead to difficulties in reflecting the intrinsic information of muscle synergy in NMF. On the contrary, the MRN could better detect highly contributing muscles in multi-muscle coordination in this study. The results indicate that MRN has a great advantage in evaluating data under dynamic steady conditions. Besides, the MRN could provide details of control at the bilateral lower limbs, unilateral lower limb, inter limbs, and single muscle levels, and has the potential to be a new tool for assessing the reticular neuromuscular control.

**Reference**

1. Naik GR, Nguyen HT. Nonnegative matrix factorization for the identification of EMG finger movements: evaluation using matrix analysis. IEEE J Biomed Health Inform. 2015;19(2):478-85.

2. Taborri J, Palermo E, Del Prete Z, Rossi S. On the Reliability and Repeatability of Surface Electromyography Factorization by Muscle Synergies in Daily Life Activities. Appl Bionics Biomech. 2018;2018:5852307.

3. Zelik KE, Scaleia VL, Ivanenko YP, Lacquaniti F. Can modular strategies simplify neural control of multidirectional human locomotion? J Neurophysiol. 2014;111(8):1686-702.

4. Torres-Oviedo G, Ting LH. Subject-specific muscle synergies in human balance control are consistent across different biomechanical contexts. J Neurophysiol. 2010;103(6):3084-98.

5. Escalona MJ, Bourbonnais D, Goyette M, Le Flem D, Duclos C, Gagnon DH. Effects of Varying Overground Walking Speeds on Lower-Extremity Muscle Synergies in Healthy Individuals. Motor Control. 2021;25(2):234-51.
